# Food sources of choline and their contribution to choline adequacy in U.S. older adults

**DOI:** 10.1016/j.jfca.2025.108484

**Published:** 2025-10-16

**Authors:** Asuka Suzuki, Jessica E. Keller, Debra K. Sullivan

**Affiliations:** Department of Dietetics and Nutrition, University of Kansas Medical Center, United States

**Keywords:** Choline, Dietary choline, Food sources, Nutrient adequacy ratio, Older adults

## Abstract

Choline is an essential nutrient, yet most Americans fail to meet the Adequate Intake (AI). This cross-sectional study investigated dietary choline sources and adequacy among 203 adults ≥ 65 years in the Midwestern U.S. Three-day food records were analyzed using the Nutrition Data System for Research, with choline intake adjusted for energy. Participants were classified into quartiles based on their choline nutrition adequacy ratio (NAR). ANOVA assessed differences in participant characteristics, while food group contributions to total choline intake were normalized within each quartile. Only 10 % of participants met the AI for choline. Choline intake was positively correlated with energy intake (*p* < 0.0001), yet choline adequacy was not associated with body mass index, waist circumference, or diet quality. Whole eggs were the largest contributor to choline intake, accounting for 22 % in the highest NAR quartile versus ~17 % in lower quartiles. These findings highlight the need for targeted dietary strategies to improve choline intake in older adults. While eggs remain the predominant source of choline, combining choline-rich, nutrient-dense foods is key to meeting the AI. For older adults with low caloric intake or following plant-based diets, particularly vegans, prioritizing choline-dense foods or considering supplementation may be necessary to maintain adequate choline status.

## 1. Introduction

Choline is an essential nutrient that plays a critical role in various physiological functions, including cell membrane formation (Eagle, 1955), the synthesis of the neurotransmitter acetylcholine (Cohen & Wurtman, 1975, 1976; Hirsch et al., 1977), lipid metabolism (Yao & Vance, 1988), and cell signaling (Exton, 1994). Choline also contributes to methylation reactions through its metabolite, betaine, which serves as a methyl donor (Niculescu & Zeisel, 2002), and also functions as an osmolyte along with glycerophosphocholine (Burg, 1995). The most abundant form of choline in the body is phosphatidylcholine (PC), which can be synthesized endogenously (Bremer & Greenberg, 1960). However, dietary intake remains the primary source of choline, as endogenous biosynthesis alone is insufficient to meet physiological demands (Ross et al., 2012).

Certain populations must ensure sufficient dietary choline intake at the recommended adequate intake (AI) or higher due to limited endogenous synthesis. This includes individuals with low estrogen levels—such as most men and postmenopausal women (Fischer et al., 2007)—as well as those with a genetic polymorphism in the phosphatidylethanolamine *N*-methyltransferase (PEMT) gene, which impairs PC synthesis (da Costa et al., 2006). Since estrogen induces PEMT (Resseguie et al., 2007), individuals with low estrogen levels or genetic variations should emphasize sufficient dietary choline intake (Fischer et al., 2010).

Despite its essential roles, the majority of the U.S. population has not met the AI recommendations (550 mg/day for men and 425 mg/day for women) (Institute of Medicine (US) Standing Committee on the Scientific Evaluation of Dietary Reference Intakes and its Panel on Folate, 1998) for decades (Freedman et al., 2024). An analysis of National Health and Nutrition Examination Survey (NHANES) data from 2005 to 2006 and 2017–2018 among adults aged 19 years and older found that mean choline intake in both men and women remained consistently 30–35 % below recommendations (2005–2006: men 409 mg/day and women 267 mg/day; 2017–2018: men 388 mg/day and women 279 mg/day) (Freedman et al., 2024). Even with dietary supplements, intake remained inadequate, with men consuming 411 mg/day in 2005–2006 and 389 mg/day in 2017–2018, and women consuming 268 mg/day and 289 mg/day, respectively (Freedman et al., 2024). This persistent inadequacy is partly due to the lack of choline fortification in food products and the low prevalence of choline supplementation in the U.S (Dietary Guidelines Advisory Committee, 2020).

Additionally, choline intake is positively correlated with total calorie intake (Chester et al., 2011). Given that the prevalence of anorexia of aging among community-dwelling older adults is estimated to be 15–30 % — due to physiological, psychological, and social challenges (Malafarina et al., 2013) — this population is also considered to be at increased risk of choline deficiency.

Choline deficiency is known to contribute to hepatic and muscle dysfunction (Fischer et al., 2007) and may impair lean mass gains in older adults (Lee et al., 2023). Moreover, growing evidence suggests that adequate choline intake may help reduce the risk of age-related cognitive decline, including neurodegenerative diseases such as Alzheimer’s disease (Niu et al., 2025; Yuan et al., 2022). Previous research indicates that choline uptake from circulating plasma into the brain declines with age (Cohen et al., 1995), suggesting that older adults may have higher choline requirements than younger individuals. Given its neuroprotective role and the observed decrease in intake associated with aging — along with lower estrogen levels in older adults — this population may particularly benefit from ensuring adequate choline intake to support cognitive function and prevent frailty.

NHANES data from 2007 to 2008 have identified the main dietary sources of choline in the U.S. population (excluding breastfed children), including dairy, eggs, meat, poultry, fish, grains, vegetables, and beverages such as beer (Chester et al., 2011). However, no studies have specifically examined dietary choline sources among older adults living in the U.S., and it remains unclear how specific foods contribute to choline intake across different levels of choline adequacy in this population. Given the physiological significance of choline and the increased susceptibility of older adults to choline deficiency, this study explores whether individuals with higher choline adequacy differ in their food sources of choline — particularly the intake of choline-dense, animal-based foods — compared to those with lower adequacy, among adults aged 65 years and older in the Midwestern U.S.

## 2. Materials and methods

This cross-sectional secondary analysis used baseline data from the Nutrition Interventions for Cognitive Enhancement (NICE) study (NCT03841539), a randomized dietary intervention conducted from February 2019 to March 2022. The study was approved by the University of Kansas Medical Center Institutional Review Board. The NICE trial was designed to compare the effects of an enhanced Mediterranean diet and a low-fat diet on cognitive function in cognitively normal adults aged 65 years and older.

### 2.1. Setting and participants

Participants were recruited from the greater Kansas City metropolitan area. This analysis used data collected at baseline, prior to randomization. Major inclusion criteria were normal cognition, defined as a Mini-Mental State Examination score ≥ 25; an Eight-Item Informant Interview to Differentiate Aging and Dementia score ≤ 2; no history of mild cognitive impairment, Alzheimer’s disease, or dementia; and no medical treatment for cognitive impairment. Exclusion criteria included currently following a Mediterranean or low-fat diet; serious medical conditions (e.g., type 1 diabetes, cancer, recent cardiac events); body mass index (BMI) < 20 or > 40 kg/m^2^; inability to manage dietary intake; current use of Warfarin; or inability to read or speak English.

### 2.2. Self-reported dietary intake

All participants completed 3-day food records (3DFRs), consisting of two weekdays, one weekend day, and daily supplement use. Study registered dietitians (RDs) provided both verbal and written instructions to ensure accurate reporting, including guidance on estimating portion sizes. During the study visit, a study RD reviewed each 3DFR with the participant to clarify any ambiguous entries. Dietary and supplement intake data were entered into the Nutrition Data System for Research (NDSR), version 2019, developed by the Nutrition Coordinating Center (NCC) at the University of Minnesota (Minneapolis, MN). Estimates of energy; macronutrients; micronutrients including choline; food group intake, and Healthy Eating Index (HEI)-2020 scores (U.S. Department of Agriculture, 2025) were derived from NDSR output files, and averaged across the three days of dietary records for each participant.

## 3. Statistical analysis

Participants with only a single-day food record or with implausible energy intakes, defined as < 500 kcal or > 3500 kcal for women and < 800 kcal or > 4000 kcal for men (Willett, 2013) were excluded.

Descriptive statistics were computed for participants’ demographic, anthropometric, and dietary intake characteristics. An ordinary least squares (OLS) regression model was used to assess the correlation between energy intake and choline consumption, with statistical significance set at p ≤ .05 to determine whether energy intake should be included as a covariate.

The NDSR categorizes foods into 135 food group identifications (IDs), which are used to classify dietary intake by source. To facilitate analysis, all authors discussed and classified these food group IDs into broader food categories (e.g., red meat) and subcategories (e.g., beef, pork, lamb), with particular consideration given to choline-rich foods. Only three participants consumed organ meats and thus organ meats were excluded prior to analysis.

To evaluate the contribution of different food groups to total dietary choline intake among individuals with varying levels of adherence to the AI for choline, we first calculated the 3-day average choline and energy intake for each participant to estimate usual intake. Daily total choline intake was assessed both overall and by food group. Choline intake was then adjusted for total energy intake (mg/1000 kcal) to account for variations in energy consumption, as energy intake was considered a potential confounder in previous studies (Chester et al., 2011; Lee et al., 2023). The Nutrient Adequacy Ratio (NAR) for choline was computed by comparing each participant’s mean choline intake to the sex-specific AI, allowing for the inclusion of all participants in a single model rather than conducting separate analyses by sex. Participants were then classified into quartiles based on their choline NAR. Quartile boundaries were determined empirically from the distribution of NAR values in the study sample (i.e., data-driven cut-points). A one-way analysis of variance (ANOVA) was conducted to assess differences in participants’ age, BMI, waist circumference, and dietary intake across quartiles. Post-hoc pairwise comparisons with Tukey’s adjustment were performed to identify specific group differences. To examine the relative contributions of different food groups to total choline intake across NAR quartiles, the mean percentage contribution of each food group was calculated within each quartile. Food group contributions were subsequently normalized within each quartile to sum to 100 %, enabling comparison of relative choline sources across varying levels of choline adequacy. Results were reported as the normalized percent contribution of broader food categories to total choline intake within each quartile of energy-adjusted choline NAR.

All statistical analyses were conducted using SAS (version 9.4, SAS Institute Inc., Cary, NC). Figures were generated using R (version 4.4.3, R Foundation for Statistical Computing, Vienna, Austria).

## 4. Results

A total of 209 participants were enrolled at baseline in the NICE study, and 203 participants (mean age: 71.4 ± 4.78 years) were included in this analysis. Participant demographic and dietary intake data are presented in Table 1. Briefly, participants were predominantly white (89.2 %), female (75.4 %), and had a BMI of 28.4 ± 4.59 kg/m^2^. Approximately 10 % of our participants met the AI for choline. Only 15 participants (7.4 %) reported using choline supplements, and their intake from supplementation was minimal (24.45 ± 26.36 mg); therefore, we did not include supplemental choline in our analysis.

**Fig. 1.**
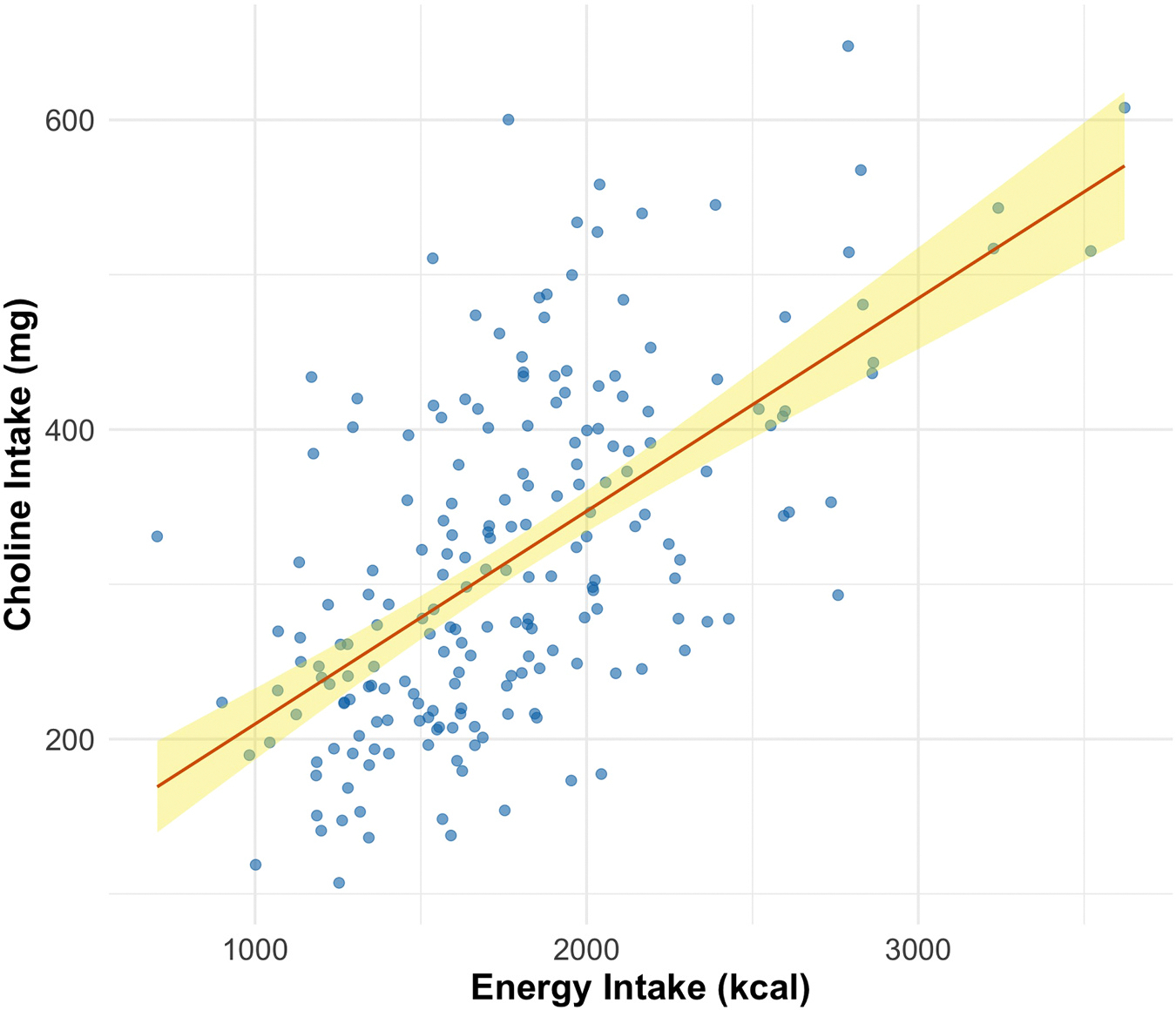
Association Between Energy Intake and Choline Intake. A scatter plot with an overlaid ordinary least squares (OLS) regression line illustrating the relationship between total energy intake (kcal) and total choline intake (mg) among participants. The shaded region represents the 95% confidence interval. β = 0.14, 95% CI: 0.11, 0.16, p = .0001.

Fig. 1 demonstrates that total energy intake is positively associated with total choline intake (β = 0.14, 95 % CI: 0.11, 0.16, *p* = .0001). Table 2 presents participants’ age, BMI, waist circumference, and dietary intake characteristics across quartiles of choline adequacy. No significant differences were observed in BMI, waist circumference, or HEI-2020 scores across quartiles. HEI-2020 scores indicated that overall diet quality aligned with the Dietary Guidelines for Americans. Participants in the lowest choline adequacy quartile (Q1; 73.4 ± 5.21 years) were significantly older than those in Q3 (70.5 ± 4.78 years, *p* = 0.01) and Q4 (70.2 ± 3.96 years, *p* < 0.01). Energy intake increased significantly from Q1 to Q4, despite quartiles being energy-adjusted. Post hoc comparisons showed no significant differences between Q1 (1480 ± 320 kcal) and Q2 (1670 ± 390 kcal, *p* = 0.10) or between Q2 and Q3 (1870 ± 400 kcal, *p* = 0.10). However, Q1 had significantly lower energy intake than Q3 (*p* < 0.0001) and Q4 (2100 ± 570 kcal, *p* < 0.0001), and Q2 had significantly lower energy intake than Q4 (*p* < 0.0001). Q4 had the highest percentage of total fat intake (41.92 ± 6.39 %), and Q1 had the highest carbohydrate intake (47.6 ± 7.28 %) and the lowest protein intake (14.6 ± 3.29 %), with significant differences observed across quartiles based on post hoc pairwise comparisons. Regarding protein sources, Q1 exhibited the highest percentage of plant-based protein intake (40.9 ± 10.94 %) and, consequently, the lowest percentage of animal-based protein intake (59.0 ± 11.04 %) compared to all other quartiles (*p* < 0.01).

**Fig. 2.**
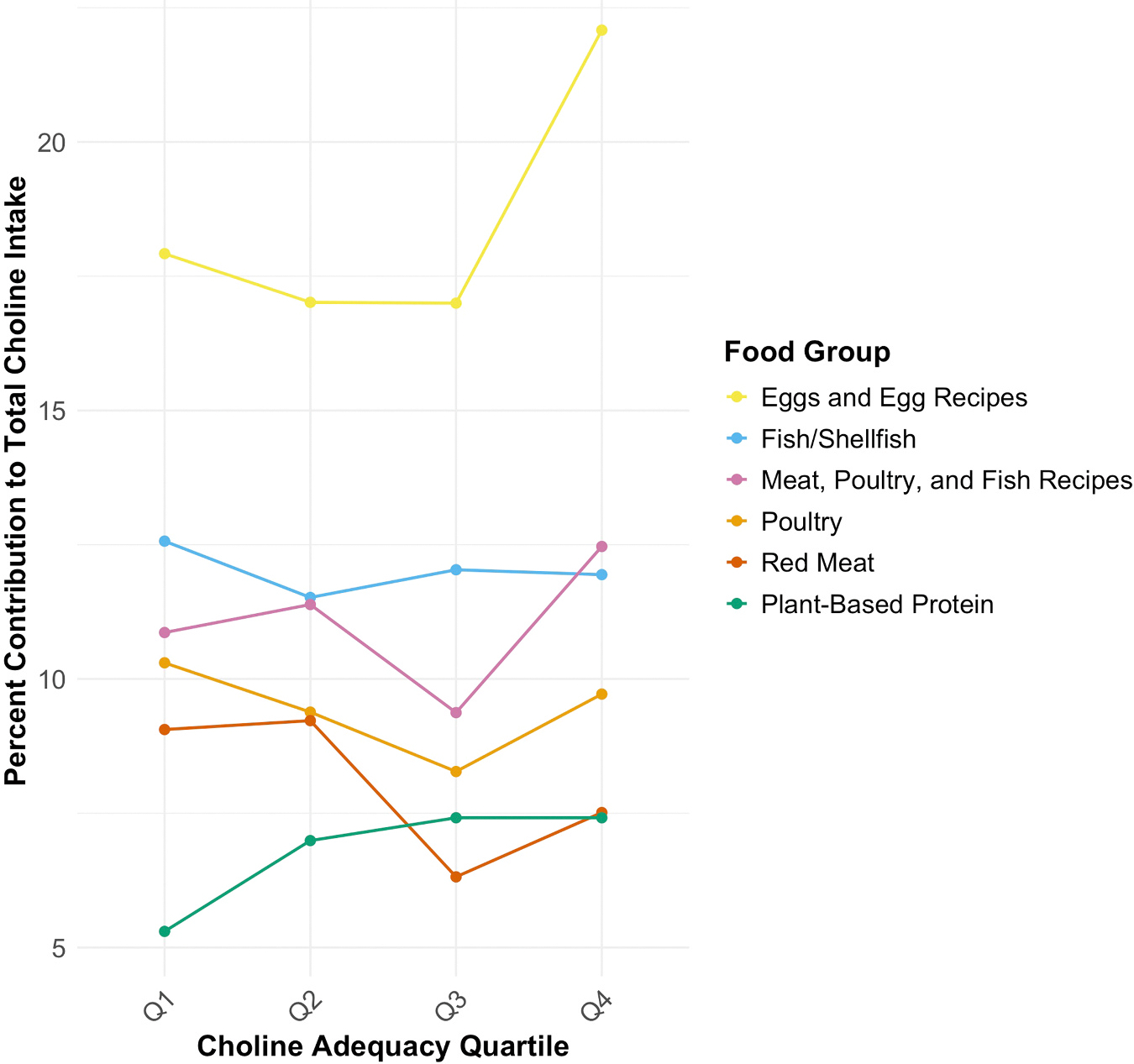
Choline Contribution by Food Group Across Quartiles of Energy-Adjusted Choline Intake. A line graph depicting the percentage contribution of different food groups to total dietary choline intake across quartiles of energy-adjusted choline adequacy ratio. Egg and egg-based dishes contributed the highest proportion of dietary choline, with a greater contribution in the highest quartile compared to lower quartiles. Other major choline sources include fish/shellfish, poultry, red meat, and plant-based protein sources.

Table 3 and Fig. 2 present the contribution of broader food categories to choline intake across quartiles. In all quartiles, whole eggs and egg-based dishes (e.g., scrambled eggs, boiled eggs, omelets, quiche) were the top contributors to choline intake. Notably, their contribution was higher in the quartile with the highest choline intake (Q4) compared to the other groups—22 % of total choline intake versus approximately 17 % in Q1–Q3. Fresh and processed animal products, including fish/shellfish, poultry, and red meat, were also major sources of choline across all quartiles, followed by plant-based sources such as legumes, grains, nuts, fruits, and vegetables.

## 5. Discussion

This study aimed to examine differences in choline sources by choline NAR to inform dietary interventions for improving choline intake among cognitively normal older adults in the Midwestern United States. Our findings contribute to a growing body of evidence indicating that many Americans, including older adults, fail to meet the AI for choline intake across the lifespan (Wallace & Fulgoni, 2016). Consistently, we observed that only 7 % of participants reported using choline supplements, and the amounts consumed were minimal. This low supplement use is in line with previous studies (Freedman et al., 2024), further reinforcing concerns about inadequate choline intake. Consistent with previous findings using NHANES 2007–2008 (Chester et al., 2011), our results showed that overall energy intake was positively associated with dietary choline intake, particularly from animal-based products. Given choline’s essential role in neurological and physiological functions, and the age-related challenges older adults face in meeting intake recommendations, identifying effective dietary strategies to help this population achieve the AI is critical.

### 5.1. Food sources of choline and dietary contributions

Our study highlights that whole eggs and egg-based recipes were the most significant contributors to dietary choline intake in this population. Given that one large whole egg provides approximately 30–35 % of the AI for choline (U.S. Department of Agriculture, 2020)I, eggs represent a highly nutrient-dense option that does not require large increases in overall food consumption. Participants with the highest choline adequacy consumed notably more choline from eggs than those in lower quartiles. This is also supported by previous research showing that egg consumers more frequently achieve choline adequacy and have nearly double the usual choline intake compared to non-egg consumers (Wallace & Fulgoni, 2017). In addition to eggs, animal-based foods such as fish/shellfish, poultry, and red meat also contributed substantially to choline intake, while plant-based sources, including legumes, played a smaller but meaningful role. Interestingly, unlike findings from NHANES 2007–2008 (Chester et al., 2011) and European cohorts (Van Parys et al., 2021), dairy contributed relatively little to choline intake in our sample.

### 5.2. Macronutrient patterns and choline adequacy

Across quartiles, choline adequacy was associated with macronutrient composition. Participants in Q1 had higher carbohydrate intake and lower fat, total protein, and animal protein intake, with an average energy intake of ~1480 kcal. In contrast, those in Q4 had higher fat, total protein, and animal protein intake and lower carbohydrate intake, with an average energy intake of ~2100 kcal. Although quartiles were classified based on energy-adjusted choline intake, total calorie intake still differed significantly. This is likely due to the higher choline density of animal-based foods, which also tend to be more calorie-dense unless consumed in lean or low-fat forms. Additionally, participants in Q1 consumed more carbohydrates and relied significantly more on plant-based protein sources than other groups. While legumes and whole grains such as quinoa contain relatively higher choline compared to other plant-based sources, their choline density is lower than that of whole eggs, meat, and fish, making it challenging to achieve adequate choline intake without exceeding caloric needs. Given that participants in Q1 were significantly older than those in Q3 and Q4, their lower intake may reflect age-related challenges such as reduced appetite or difficulty consuming larger food quantities. These findings have important dietary implications. Individuals consuming lower total calories (e.g., 1400–1500 kcal/day) need to prioritize choline-dense foods such as whole eggs to meet the AI.

### 5.3. Implications for dietary recommendations and future research

Since higher choline adequacy was associated with greater total energy intake and increased animal protein consumption in our sample, and prior studies have shown that higher energy and animal protein intake are correlated with higher BMI (Arini et al., 2025; Bricarello et al., 2025), greater waist circumference (Arini et al., 2025), or lower diet quality (e.g., lower HEI scores (Hoy et al., 2022)), one might expect similar patterns here. However, our results showed no significant differences in BMI, waist circumference, or HEI-2020 across quartiles. Although the association between choline and body composition remains inconclusive (Gao et al., 2018; Gao et al., 2016), our findings suggest that higher choline intake does not necessarily correlate with poorer diet quality or adverse body composition outcomes. That said, the average HEI-2020 score in our sample indicates room for improvement. Therefore, future research should examine strategies for achieving choline adequacy through dietary patterns that balance nutrient-dense animal products with a variety of nutrient-rich plant-based choline sources, in order to support overall diet quality.

Older adults with lower calorie intake or those consuming predominantly plant-derived diets — especially vegan diets that exclude all animal products — should prioritize choline-dense foods to meet their nutritional needs. If achieving the AI for choline through diet alone proves challenging, supplementation may be necessary to help individuals meet recommended intake levels without excessive caloric intake. To address widespread inadequate choline intake, we also support suggestions from previous research (Wallace & Fulgoni, 2016) that fortifying widely available and cost-effective foods, such as grains and dairy, may be necessary. Given the high prevalence of choline inadequacy in the general population, this approach warrants further exploration in public health and policy discussions.

### 5.4. Strength and limitations

One of this study’s key strengths is the use of 3DFRs, which offer a more accurate snapshot of habitual nutrient intake than single-day assessments. Because dietary patterns can vary considerably from day to day, collecting multiple days of intake data helps mitigate measurement error associated with self-reported intake. Additionally, our community-based sample of 203 adults aged 65 years and older provides important insights into the real-world eating behaviors of an often-underrepresented population. By analyzing choline intake across quartiles, we were able to explore variations in dietary patterns among participants with different levels of intake, illuminating food choices that may help older adults meet the AI for this nutrient.

There are, however, some limitations to this study. First, despite the advantages of repeated food records, self-reported dietary data are vulnerable to recall errors, social desirability and recall bias, and misreporting. Second, while the sample size is robust, the generalizability of these findings to broader populations of older adults in other regions may be limited, as our sample was predominantly white, non-Hispanic, female, and highly educated. Third, mixed dishes in participants’ diets may have obscured the precise contribution of individual ingredients; this can lead to minor inaccuracies in calculating choline intake. Lastly, we did not validate reported choline consumption with biochemical markers (e.g., plasma choline levels), limiting our ability to confirm whether the observed dietary patterns reflect actual physiological choline status.

Despite these limitations, the detailed nature of the three-day food record methodology, the community-based sample, and the targeted examination of choline intake strengthen the relevance and rigor of our findings. This approach offers valuable perspectives on dietary patterns that could guide future nutrition interventions aimed at older adults, an especially important investigation in an aging U.S. population.

## 6. Conclusions

Taken together, these findings highlight the continued need for targeted, feasible dietary strategies to improve choline intake among older adults. Given choline’s essential role in supporting cognitive health and preventing frailty, ensuring that older adults meet the AI is of particular importance. Emphasizing choline-rich, nutrient-dense foods — such as whole eggs — while also identifying strategies to incorporate these foods into the daily diet to promote overall diet quality and well-being will be essential for the aging population. In light of the high prevalence of choline inadequacy in the U.S. — particularly among older adults with low caloric intake or those who predominantly consume plant-derived diets, especially those excluding all animal products — strategies such as food fortification and supplementation warrant further investigation.

## Figures and Tables

**Table 1:** Baseline demographics *n* = 203.

Age (years)	71.4 ± 4.78

**Sex**	
**Female**	151 (74.4 %)
**Male**	52 (25.6 %)
**Education**	15.9 ± 3.57
**Race**	
**Black or African American**	18 (8.9 %)
**White**	183 (90.2 %)
**Other**	2 (0.9 %)
**Ethnicity**	
**Hispanic or Latino**	4 (2.0 %)
**Not Hispanic or Latino**	199 (98.0 %)
**BMI (kg/m^2^)**	28.4 ± 4.59
**Healthy Eating Index (HEI)–2020**	63.9 ± 8.15
**Met AI for choline**	20 (9.9 %)
**Choline Supplement Use (Yes)**	15 (7.4 %)
**Choline Supplement Intake (mg/day)**	24.45 ± 26.36

Values are presented as mean ± standard deviation (SD) for continuous variables and as frequency (percentage) for categorical variables.

**Table 2:** Participant characteristics across choline adequacy quartiles (*n* = 203).

Variable	Q1 (≤ 51.3 %)	Q2 (51.4–67.0 %)	Q3 (67.1–88.2 %)	Q4 (> 88.2 %)	*p* -value

**Age, year**	73.4 ± 5.21	71.6 ± 4.60	70.5 ± 4.78	70.2 ± 3.96	< 0.01
**BMI, (kg/m^2^)**	28.6 ± 4.58	28.0 ± 4.45	28.4 ± 4.19	28.8 ± 5.17	0.83
**Waist, cm**	97.1 ± 13.06	96.7 ± 15.41	98.6 ± 13.45	99.6 ± 15.83	0.74
**Energy, kcal**	1480 ± 320	1670 ± 390	1870 ± 400	2100 ± 570	< 0.0001
**Choline, mg**	196.6 ± 38.54	263.2 ± 35.43	346.3 ± 50.04	460.1 ± 68.12	< 0.0001
**% CHO**	47.6 ± 7.28	43.7 ± 7.62	43.1 ± 6.90	40.4 ± 8.01	< 0.0001
**% Fat**	37.9 ± 7.69	38.2 ± 6.87	37.8 ± 6.48	41.9 ± 6.39	< 0.01
**% Total Protein**	14.6 ± 3.29	16.6 ± 2.89	16.8 ± 3.53	17.5 ± 3.52	< 0.001
**% Animal Protein**	59.0 ± 11.04	64.9 ± 9.91	64.8 ± 9.97	66.9 ± 11.66	< 0.01
**% Plant Protein**	40.9 ± 10.94	35.0 ± 10.06	35.2 ± 9.97	33.1 ± 11.66	< 0.01
**HEI–2020**	60.8 ± 12.40	62.6 ± 11.17	61.4 ± 10.7	62.1 ± 10.7	0.87

Comparisons were performed using one-way analysis of variance (ANOVA). Values are presented as mean ± standard deviation (SD).

**Table 3:** Percent contribution of foods to choline intake by quartile (*n* = 203).

Food categories^a^	Q1 (≤51.3 %)	Q2 (51.4–67.0 %)	Q3 (67.1–88.2 %)	Q4 (>88.2 %)

Eggs and egg recipes	17.9	17.0	17.0	22.1
Fish and shellfish	12.6	11.5	12.0	11.9
Meat, poultry, and fish recipes^b^	10.9	11.4	9.4	12.5
Poultry (fresh or processed)	10.3	9.4	8.3	9.7
Red meat (fresh or processed)^c^	9.1	9.2	6.3	7.5
Plant-based protein^d^	5.3	7.0	7.4	4.1
Grain^e^	2.6	2.6	2.3	2.1
Grain-based recipes^f^	4.5	3.9	13.1	4.3
Commercial entrees and dinners	3.3	6.3	5.8	7.4
Dairy^g^	3.2	3.0	2.4	2.6
Nuts and nut butters	3.2	3.3	2.7	3.0
Sweets^h^	2.7	2.6	2.7	2.4
Fruits and vegetables	2.9	2.4	1.9	2.2

a Food categories not listed, including soups and gravy, milk- or soy-based meal replacements and supplements, grain-based snacks, beverages, fats, candy, sugar, honey, jelly, and other miscellaneous foods, each contribute less than 4 % of total choline intake

b Includes red meat, poultry, and fish-based mixed dishes like meatloaf, stew, barbecue, chicken pot pie, pizza with meat, etc.

c Includes beef, pork, lamb, game (venison or deer), or combination red meat

d Includes legume and plant-based meat substitutes

e Includes bread, baked products, cereals, granolas, grain products such as oatmeal, rice, pasta, etc.

f Includes grain-based mixed dishes like burritos, sandwiches, pizza without meat, etc.

g Includes cow’s milk, yogurt, cheese, cream, ice cream etc.

h Includes cakes, cookies, pies, and puddings

## Data Availability

Data will be made available on request.
